# Dynamics of plant DNA replication based on PCNA visualization

**DOI:** 10.1038/srep29657

**Published:** 2016-07-15

**Authors:** Ryohei Yokoyama, Takeshi Hirakawa, Seri Hayashi, Takuya Sakamoto, Sachihiro Matsunaga

**Affiliations:** 1 Department of Applied Biological Science, Faculty of Science and Technology, Tokyo University of Science, 2641 Yamazaki, Noda, Chiba 278-8510, Japan

## Abstract

DNA replication is an essential process for the copying of genomic information in living organisms. Imaging of DNA replication in tissues and organs is mainly performed using fixed cells after incorporation of thymidine analogs. To establish a useful marker line to measure the duration of DNA replication and analyze the dynamics of DNA replication, we focused on the proliferating cell nuclear antigen (PCNA), which functions as a DNA sliding clamp for replicative DNA polymerases and is an essential component of replisomes. In this study we produced an *Arabidopsis thaliana* line expressing PCNA1 fused with the green fluorescent protein under the control of its own promoter (*pAtPCNA1::AtPCNA1-EGFP*). The duration of the S phase measured using the expression line was consistent with that measured after incorporation of a thymidine analog. Live cell imaging revealed that three distinct nuclear localization patterns (whole, dotted, and speckled) were sequentially observable. These whole, dotted, and speckled patterns of subnuclear AtPCNA1 signals were indicative of the G1 or G2 phase, early S phase and late S phase, respectively. The results indicate that the *pAtPCNA1::AtPCNA1-EGFP* line is a useful marker line for visualization of S-phase progression in live plant organs.

DNA replication is the process by which genomic information of living organisms is copied. Regulation of the timing of DNA replication is required for developmental and differentiation processes^1^,^2^,^3^. Generally, euchromatin is predominantly replicated in the early S phase, whereas heterochromatin is replicated in the late S phase^4^. DNA replication in *Arabidopsis thaliana* is also classified into early and late replication, based on the formation of replicons, which were indicated by tiling microarray analyses^5^. Imaging analyses using a thymidine analog, 5-ethynyl-2′-deoxyuridine (EdU), revealed that the spatiotemporal patterns of DNA replication were three-dimensionally altered in nuclei of root tip cells of *A. thaliana*^6^ and maize^7^,^8^. However, EdU is not applicable for live cell imaging because fixation of cell nuclei is required for detection of incorporated EdU^9^. A dual marker line, Cytrap, was established using a G2/M marker and an S/G2 marker but could not determine the duration of the S phase^10^. A marker line for monitoring the progression of DNA replication in living tissues of plants has not been reported.

In order to establish a line for live cell imaging of DNA replication in plant organs, we focused on the landmark protein of DNA replication, proliferating cell nuclear antigen (PCNA), which is characterized as a sliding clamp for replicative DNA polymerases and is an essential component of replisomes^11^,^12^,^13^. With the induction of DNA replication, DNA synthesis is initiated in both leading and lagging strands by the DNA polymerase α/primase complex, which synthesizes short template RNA required for initiation of DNA replication^14^,^15^. Subsequently, PCNA is loaded onto the DNA and functions as a scaffold for DNA polymerase δ, ε and other proteins required for DNA replication^16^. PCNA enables the exchange of DNA polymerase α with other replicative polymerases for progression of DNA replication^11^. In yeast and animal cells, nuclear localization of PCNA in the S phase exhibits a very distinct pattern. Small foci of PCNA were observed throughout the whole nucleus in the early S phase, whereas large foci of PCNA were observed in the perinuclear and perinucleolar regions in the late S phase^17^,^18^,^19^. To test whether PCNA protein may be a useful S-phase marker in plants, we produced a transgenic line of *A. thaliana* expressing AtPCNA1-EGFP under the control of its own promoter. We observed that AtPCNA1 showed three distinct patterns of subnuclear localization in interphase nuclei; i.e., whole, dotted, and speckled signal patterns were observed in nuclei at the G1 or G2 phase, early S phase and late S phase, respectively. In the present study we established a marker line useful for analyzing S-phase progression in live plant organs.

## Results

### AtPCNA1 exhibited three distinct nuclear localization patterns in interphase nuclei

**Figure 1:**
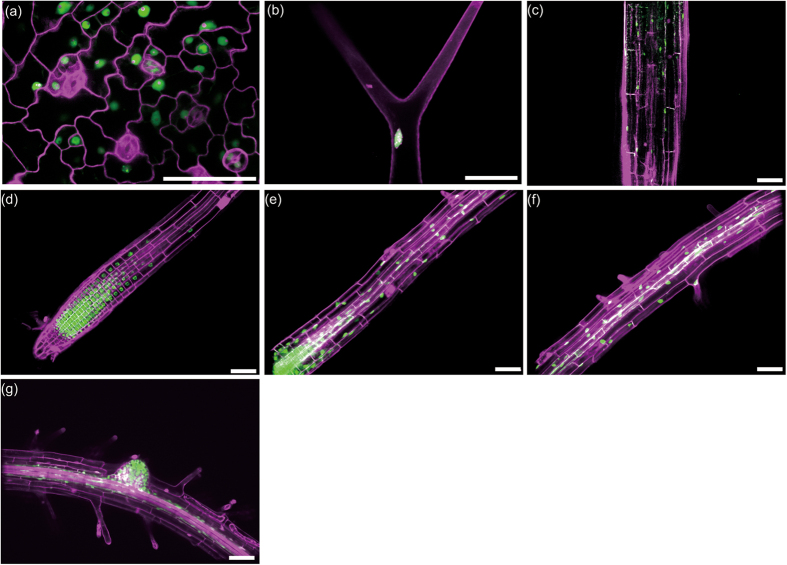
Expression pattern of AtPCNA1-EGFP in *A. thaliana*. (**a**,**b**) Leaf epidermis (**a**) and trichome on a leaf (**b**) of plants expressing AtPCNA1-EGFP at 11 days after sowing (DAS). (**c**–**g**) Hypocotyl (**c**), root meristematic zone (**d**), root elongation zone (**e**), root differentiation zone (**f**), and a lateral root primordium (**g**) of plants expressing AtPCNA1-EGFP at 12 DAS. Cell walls stained with propidium iodide and AtPCNA1-EGFP signals are shown in magenta and green, respectively. Bars = 50 μm.

**Figure 2:**
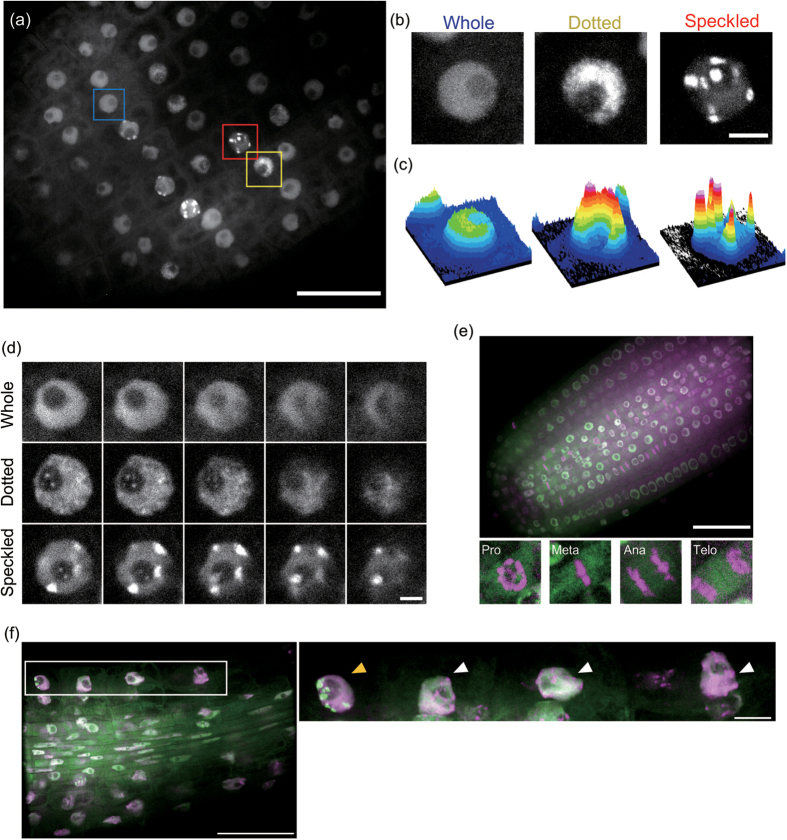
Subcellular localization and dynamics of AtPCNA1-EGFP signals in *A. thaliana* roots. (**a**) AtPCNA1-EGFP signals in the root meristematic zone. Blue, yellow and red boxes indicate nuclei with whole, dotted, and speckled patterns of AtPCNA1-EGFP signals, respectively. Bar = 20 μm. (**b**) Magnified images of nuclei with whole, dotted, and speckled patterns of AtPCNA1-EGFP signals. These are Z-stack maximum-intensity projection images. Bar = 3 μm. (**c**) Surface plots of fluorescence images shown in (**b**). (**d**) Optical slices of each AtPCNA1 signal pattern. Images show a series of *Z*-slices at each 0.5 μm interval. Bar = 3 μm. (**e**) Subcellular localization of AtPCNA1 (green) in nuclei stained with DAPI (magenta) in the root meristematic zone. AtPCNA1 was localized in interphase nuclei, but not mitotic chromosomes at prophase (Pro), metaphase (Meta), anaphase (Ana) and telophase (Telo). Bar = 50 μm. (**f**) Subcellular localization of AtPCNA1 (green) in nuclei stained with DAPI (magenta) in the root elongation zone. Chromocenters were detected in the cells that showed the whole pattern of AtPCNA1-EGFP signals (white arrowheads), but not in cells exhibiting the speckled pattern of AtPCNA1-EGFP signals (yellow arrowhead). The right panel is a high-magnification image of the left panel. Bar = 50 μm (left panel) and 10 μm (right panel).

**Figure 3:**
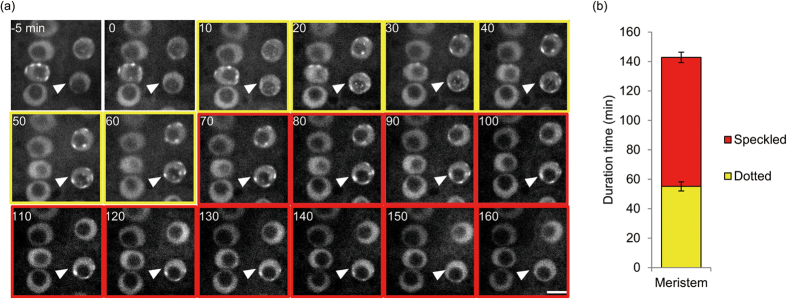
Live imaging of AtPCNA1-EGFP through the cell-cycle progression. (**a**) Images taken at 5 min intervals in the meristematic zone of a root expressing AtPCNA1-EGFP. The pattern of AtPCNA1 signals changed sequentially from whole to dotted, speckled and whole through the cell-cycle progression. The numbers indicate duration from time 0 in minutes. The yellow and red outer frames indicate that signals in the nucleus indicated by a white arrowhead exhibit dotted and speckled patterns, respectively. Bar = 5 μm. (**b**) Duration of dotted and speckled patterns of AtPCNA1-EGFP signals (*n* = 7 for dotted pattern, *n* = 8 for speckled pattern). Error bars denote the SE.

To investigate the expression pattern of AtPCNA1, we transformed the genomic sequence of *AtPCNA1* ligated with the *GFP* cDNA sequence under the control of the native promoter into *A. thaliana*. We specifically observed AtPCNA1-EGFP signals in nuclei of the epidermal cells of leaves, trichomes, hypocotyls, the meristematic, elongation and differentiation zones of roots, and lateral root primordia (Fig. 1a–g). These results indicated that AtPCNA1 is specifically expressed in nuclei of diverse tissues of *A. thaliana*. We observed the subcellular localization of AtPCNA1-EGFP signals in roots (Fig. 2a). Remarkably, AtPCNA1 signals were classifiable into three distinct patterns, namely whole, dotted, and speckled patterns (Fig. 2b). The whole pattern was characterized by uniform localization of AtPCNA1 in the nucleoplasm. The dotted pattern was characterized by many dot-like signals with 1.1- to 1.4-times higher maximum intensity compared with signals in the whole pattern. The speckled pattern was characterized by speckle signals with a more than 1.4-fold increase in maximum intensity compared with signals in the whole pattern. The *Z*-projection images were prepared from 23 optical sections at regular 0.2 μm intervals in each pattern. The surface plot analyses confirmed that the maximum intensity of AtPCNA1 signals in the dotted pattern was higher than that of the whole pattern (Fig. 2c). The speckled pattern was characterized by localization of completely separated AtPCNA1 signals. When the optical sections were prepared by confocal microscopy, the signals were observed in the perinuclear and perinucleolar regions (Fig. 2d). When nuclei and chromosomes were stained with 4′,6-diamidino-2-phenylindole (DAPI), AtPCNA1 was not localized to condensed chromatin and mitotic chromosomes (Fig. 2e). DAPI-positive subnuclear regions, chromocenters, were detected in the nuclei with the whole pattern of AtPCNA1 signals, but were not observed in the case of the speckled signal pattern (Fig. 2f). To investigate how the three patterns of AtPCNA1 signals sequentially changed through the cell cycle, we performed time-lapse imaging of AtPCNA1-EGFP roots in the meristematic zone. AtPCNA1 signals dynamically changed through the cell cycle. AtPCNA1 signals sequentially changed from whole to dotted, speckled and then whole again through the cell cycle over the course of 2.5–3 h (Fig. 3a). When the duration of each pattern of AtPCNA1-EGFP signals was measured, the duration of the dotted and speckled patterns was approximately 60 and 80 min, respectively (Fig. 3b), indicating that the subnuclear localization of AtPCNA1 dynamically changed in the root meristematic zone.

### AtPCNA1 signal patterns are indicators of the progression of DNA replication

**Figure 4:**
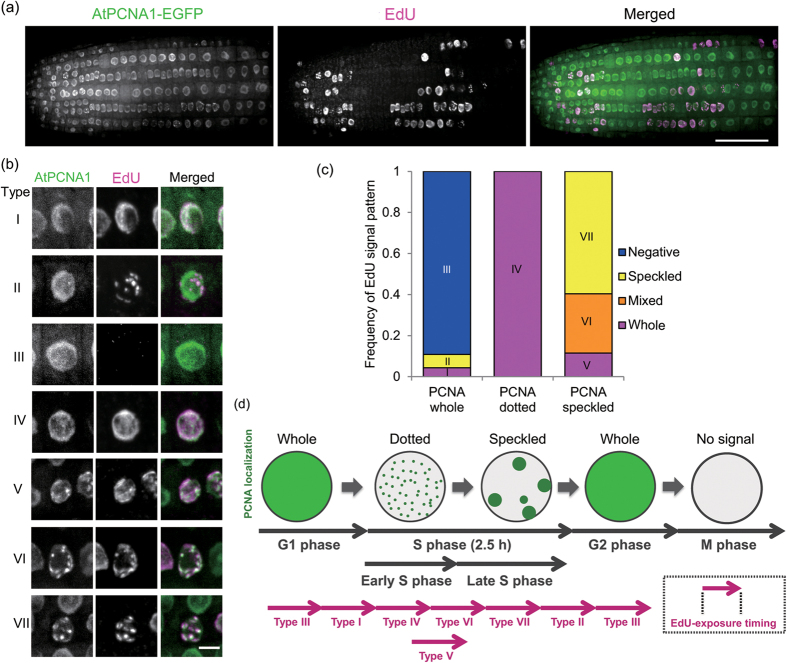
Relationship between subnuclear localization patterns of AtPCNA1 and DNA replication. (**a**) EdU signals (magenta) in the meristematic zone of a root expressing AtPCNA1-EGFP (green). Bar = 50 μm. (**b**) Types of subnuclear localization patterns of AtPCNA1-EGFP and EdU signals. The classification of types is shown in Supplementary Table 1. Bar = 5 μm. (**c**) Frequency of each EdU signal pattern in nuclei with whole, dotted and speckled patterns of AtPCNA1 signals in epidermal cells of the root meristematic zone (*n* = 231 [whole], *n* = 24 [dotted] and *n* = 52 [speckled] nuclei from three individual plants). The blue graph (Type III) shows the frequency of EdU-negative cells. Yellow, orange and purple graphs indicate the frequency of the speckled, mixed and whole patterns of EdU signals, respectively. (**d**) Timeline of AtPCNA1 dynamics in the cell-cycle progression. Whole, dotted, and speckled patterns of AtPCNA1 signals are indicative of the G1 or G2 phase, early S phase and late S phase, respectively.

To analyze the relationship between the subnuclear localization pattern of AtPCNA1 and the progression of DNA replication, we visualized newly synthesized DNA with a thymidine analog, 5-ethynyl-2′-deoxyuridine (EdU)^9^. After EdU was incorporated into seedlings for 30 min, EdU signals were detected in AtPCNA-positive nuclei (Fig. 4a). Although AtPCNA1-EGFP signals were slightly quenched, we could discriminate between whole and dotted patterns (Supplementary Figure S1a). The signal patterns were classified into seven representative merged patterns: Type I, whole PCNA1 and EdU signals; Type II, whole PCNA1 and speckled EdU signals; Type III, whole PCNA1 signals without EdU signals; Type IV, dotted PCNA1 and whole EdU signals; Type V, speckled PCNA1 and whole EdU signals; Type VI, speckled PCNA1 and mixed EdU signals; and Type VII, speckled PCNA1 and EdU signals (Fig. 4b, Supplementary Table S1). Next, we analyzed these merged patterns quantitatively in the epidermal cell layer of the meristematic zone of roots. The frequency of the whole, dotted, and speckled patterns of AtPCNA1 signals was approximately 70%, 10% and 20%, respectively (Supplementary Figure S1b). We calculated the frequency of EdU-positive nuclei that exhibited each pattern of AtPCNA1 signals (Fig. 4c). We previously reported that the whole and speckled patterns of EdU signals indicate the early and late S phase, respectively^6^. All nuclei with the dotted pattern of AtPCNA1 signals exhibited the whole pattern of EdU signals (Type IV), indicating that Type IV nuclei were in the early S phase. Most nuclei with the speckled pattern of AtPCNA1 signals showed mixed (Type VI) or speckled (Type VII) patterns of EdU signals. The EdU mixed pattern, which was an intermediate pattern between the whole and speckled patterns, was indicative of nuclei between the early and late S phase, and nuclei with the speckled pattern of AtPCNA1 signals were in the late S phase. Nuclei (Type III) with the whole pattern of AtPCNA1 signals without EdU signals were at the G1 or late G2 phase. Nuclei (Type II) with the whole pattern of AtPCNA1 signals and the speckled pattern of EdU signals were in the early G2 phase (Fig. 4c). These results revealed that the whole, dotted, and speckled patterns of AtPCNA1 signals indicate that cells are in the G1 or G2, early S phase and late S phase, respectively (Fig. 4e).

We previously reported that the duration of the S phase and cell cycle in the meristematic zone of roots is 3 h and 17 h, respectively^6^. Cytrap showed that the duration of the complete cell cycle is 16 h^10^. We compared the ratio of the number of nuclei with the dotted and speckled patterns of AtPCNA1 signals to total nuclei and the ratio of S-phase duration to cell-cycle duration. The ratio of AtPCNA1 signals was almost equal to that previously reported (Supplementary Figure S1c).

### Inhibitors of cell-cycle progression influence AtPCNA1 signal patterns

**Figure 5:**
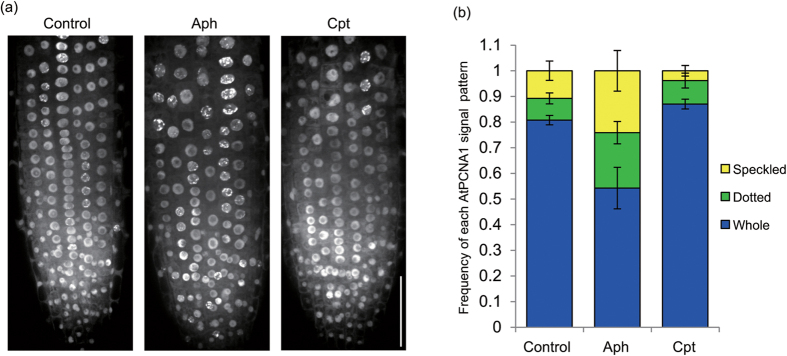
Effect of inhibitors of cell-cycle progression on the frequency of AtPCNA1 localization patterns. (**a**) AtPCNA1 signals in the meristematic zone of roots treated for 24 h with 12 μg/mL aphidicolin (Aph) or 5 nM camptothecin (Cpt). Bar = 50 μm. (**b**) Frequency of each AtPCNA1 signal pattern in epidermal cells of the root meristematic zone. *n* ≥ 287 nuclei from 3–5 individual plants. Error bars indicate the SD.

To further confirm that each AtPCNA1 signal pattern was indicative of a specific phase of the cell cycle, we analyzed the influence of cell-cycle inhibitors on the pattern of AtPCNA1 signals in the root meristematic zone. When treated with aphidicolin, an inhibitor of DNA polymerase α, which catalyzes the synthesis of template RNA required for initiation of DNA replication^20^, the dotted and speckled patterns of AtPCNA1 signals were observed at high frequency (Fig. 5a,b). On the other hand, after treatment with camptothecin, an inhibitor of topoisomerase I, which is involved in unwinding double-strand DNA to single-strand DNA required for initiation of DNA replication^21^, the whole pattern of AtPCNA1 signals was observed at high frequency (Fig. 5a,b).

## Discussion

In this study, we first analyzed plant PCNA dynamics during DNA replication. The subnuclear localization pattern of PCNA signals enabled determination of the G1 to S phase transition, and S to G2 phase transition. Moreover, on the basis of the signal patterns, the S phase was classifiable into early and late S phase. Although the whole pattern of AtPCNA1 signals does not exhibit the S phase, EdU signals were weakly detected in the nuclei that showed the whole pattern of AtPCNA1 signals (Type I) (Fig. 4c). There are two possible explanations of the Type I signal pattern. First, the dotted pattern of AtPCNA1 signals may not be distinguishable from the whole pattern because of quenching of AtPCNA1-EGFP signals after the procedure for EdU detection. Second, nuclei that showed the whole pattern of AtPCNA1 signals immediately before the dotted pattern may already have entered the S phase.

The duration from the appearance of the speckled pattern of AtPCNA1 signals in a nucleus to its reappearance was 16.2 ± 0.4 h (*n* = 3). This suggests that the duration of the cell cycle is approximately 16 h in the root meristematic zone, which is consistent with previous reports^22^. The ratio of dotted and speckled patterns of AtPCNA1 signals was compared with the ratio of nuclei at the S phase as reported previously^6^, and consequently the ratio of AtPCNA1 signals was equal to previously reported ratios (Supplementary Figure S2). These results strongly suggest that the *pAtPCNA1::AtPCNA1-EGFP* line is a useful marker line for visualization of S-phase progression.

We revealed that nuclei that showed the dotted pattern of AtPCNA1 signals were in the early S phase (Fig. 4). In eukaryotes, the nuclear structures that are formed by association of neighboring replication origins, called replication foci, were observed during DNA replication progression^23^. Proteins that are needed for DNA replication progression are accumulated in replication foci, and are visualized by immunostaining of PCNA or using a fluorescent protein in human and yeast cell cultures^17^,^18^,^19^. The dotted signals probably indicate replication foci, which are also observable in *Allium cepa* and *Zea mays* by immunostaining of PCNA or EdU incorporation^7^,^24^.

We revealed that nuclei that showed the speckled pattern of AtPCNA1 signals were in the late S phase (Fig. 4). Signals were observed in the perinuclear and perinucleolar regions, which suggests that the nuclear location of replication of heterochromatic regions such as centromeric regions is the same as in animals^5^. Chromocenters, which are condensed chromatin segments, were observed in the nuclei that exhibited the whole AtPCNA1 signal pattern, but were not observed in nuclei that showed the speckled pattern of AtPCNA1 signals (Fig. 2f). These results suggest that heterochomatic regions are decondensed structures in the late S phase.

The frequency of each AtPCNA1 signal pattern was analyzed after treatment with well-known inhibitors of cell-cycle progression (Fig. 5a,b). Following treatment with aphidicolin, the frequency of the AtPCNA1 dotted and speckled patterns increased, and that of the whole pattern was reduced. These results suggest that S phase progression is suppressed by aphidicolin treatment. Based on a S phase duration of 3 h and complete cell cycle of 16 h, almost all cells in the root meristematic zone were considered to be arrested in the S phase after aphidicolin treatment for 24 h. However, the root elongated after treatment with aphidicolin (Supplementary Figure S2). Endoreduplication, in which cells repeatedly replicate their DNA without cytokinesis, plays an important role in the expansion and elongation of organs in many plant species^6^,^25^,^26^. In tobacco (*Nicotiana tabacum*) BY-2 cells, endoreduplication and cell expansion progress even after inhibition of DNA polymerase α by treatment with aphidicolin^27^,^28^. These results suggest that another DNA polymerase, in addition to DNA polymerase α, is expressed in *A. thaliana*. An alternative possibility is that aphidicolin is ineffective at inhibiting DNA polymerase α in *A. thaliana*. After treatment with camptothecin, the frequency of the whole pattern of AtPCNA1 signals was increased, whereas that of the dotted and speckled patterns was reduced (Fig. 5a,b). These findings indicated that cells were prevented from entering the S phase after camptothecin treatment.

Visualization of DNA replication is necessary for elucidation of temporospatially developing mechanisms in plants. Elucidation of plant PCNA dynamics will help to understand the machinery of tissue-specific DNA replication. Moreover, development of a line for imaging DNA replication will be useful to enable screening for lead compounds from agricultural chemicals, determination of the points of action of inhibitors, and identification of the phenotype of mutants.

## Methods

### Plant material and growth conditions

*Arabidopsis thaliana* accession Columbia expressing *pAtPCNA1::AtPCNA1-EGFP* was constructed by the following procedures. The *AtPCNA1* gene was amplified by PCR and subcloned into the pENTR/D-TOPO vector for entry into the Gateway System (Thermo Fisher Scientific, http://www.thermofisher.co.jp/), then recombined into the pGWB504 binary vector^29^ using LR Clonase II (Thermo Fisher Scientific). The PCR primers used are listed in Supplementary Table S2. The floral dip method was used to introduce the constructed vector into *A. thaliana* plants. Seeds were germinated on Murashige and Skoog (MS) gellan gum plates containing 1/2 MS salts, 1% sucrose and 1% gellan gum at pH 5.8. The plated seeds were stratified at 4 °C for 1 day and then incubated in a plant growth chamber CLH-301 (TOMY, http://bio.tomys.co.jp/) at 22 °C with a 16 h/8 h (light/dark) photoperiod.

### Time-lapse imaging

At 7 days after sowing (DAS), seedlings expressing *pAtPCNA1::AtPCNA1-EGFP*, which were grown on 1/2 MS gellan gum medium in a glass-bottom dish, were observed under an inverted fluorescence microscope (IX81, Olympus, http://www.olympus-lifescience.com/ja/) equipped with a confocal scanning unit (CSU-X1, Yokogawa, http://www.yokogawa.co.jp/) and a sCMOS camera (Neo 5.5 sCMOS, ANDOR, http://www.andor.com/). Z-stack images were acquired every 30 or 5 min for 17.5 h or 3 h, respectively.

### EdU incorporation assay

Detection of EdU was performed with the Click-iT Plus EdU Alexa Fluor 594 Imaging Kit (Thermo Fisher Scientific) following the manufacturer’s guidelines. For EdU incorporation, 7 DAS seedlings expressing pAtPCNA1::AtPCNA1-EGFP grown on 1/2 MS gellan gum plates were transferred to liquid 1/2 MS medium containing 10 μM EdU for 30 min. Seedlings were fixed for 20 min in 4% (w/v) paraformaldehyde in PBS (pH 7.4), washed twice with PBS and placed in 0.5% (v/v) TritonX-100 in PBS. After 20 min, samples were washed with PBS twice and incubated in the Click-iT reaction cocktail for 30 min in the dark. The Click-iT reaction cocktail was removed and samples were washed with PBS once. The samples were observed under an inverted fluorescence microscope (IX81, Olympus), which included the laser (405 nm for DAPI, 488 nm for EGFP and 561 nm for Alexia Fluor 555 for EdU detection), equipped with a confocal scanning unit (CSU-X1, Yokogawa) and a sCMOS camera (Neo 5.5 sCMOS, ANDOR). Maximum-intensity projections along the Z-axis (0.5 μm per stack) are shown in Fig. 4. Meristematic epidermal cells were used for analysis of merged signal patterns for AtPCNA1 and EdU. Images were analyzed with Image J software (US National Institutes of Health, Bethesda, MD, USA, http://imagej.nih.gov/ij/). The root meristematic zone and elongation zone were distinguished on the basis that the distance between adjacent nuclei in the elongation zone is more than twice that of the meristematic cells.

### Chemical treatment

For the treatments with aphidicolin (Wako, http://www.wako-chem.co.jp/) and camptothecin (Thermo Fisher Scientific), 4 DAS seedlings pre-incubated on 1/2 MS gellan gum plates were transferred to control medium and medium supplemented with the indicated drugs. The frequency of each AtPCNA1 signal pattern was calculated in root meristematic epidermal cells in each fraction. The root meristematic zone was defined as described above.

### Other imaging procedures

For DAPI staining, 5 DAS seedlings expressing *pAtPCNA1::AtPCNA1-EGFP* were fixed for 20 min in 4% (w/v) paraformaldehyde in PBS (pH 7.4). After washing with PBS twice, samples were incubated in 0.5% TritonX-100 in PBS for 5 min at room temperature. This step was repeated twice, then samples were washed with PBS once and stained with CyStain staining solution (Partec, http://www.sysmex-partec.com/), which was diluted 4-fold with PBS for 3 min. After three washes with PBS, samples were observed under an inverted fluorescence microscope as described above. For confirmation that the AtPCNA1-GFP line is a useful marker line for visualization of S-phase progression, the number of nuclei that exhibited the AtPCNA1 dotted and speckled patterns was scored, and the ratio of nuclei showing the AtPCNA1 dotted and speckled patterns to total nuclei stained with DAPI was calculated and compared with the ratio of S-phase length to cell-cycle length previously reported^6^,^10^. The root meristematic zone was defined as described above. For visualization of the cell wall, *A. thaliana* seedlings were incubated in propidium iodide diluted 500 times with water for 4 min.

## Additional Information

**How to cite this article**: Yokoyama, R. *et al*. Dynamics of plant DNA replication based on PCNA visualization. *Sci. Rep.* **6**, 29657; doi: 10.1038/srep29657 (2016).

## Supplementary Material

Supplementary Information
