# Corrigendum: Dynamics of plant DNA replication based on PCNA visualization

**DOI:** 10.1038/srep40831

**Published:** 2017-01-13

**Authors:** Ryohei Yokoyama, Takeshi Hirakawa, Seri Hayashi, Takuya Sakamoto, Sachihiro Matsunaga

Scientific Reports 6: Article number: 2965710.1038/srep29657; published online: 07 15 2016; updated: 01 13 2017

In the original version of this Article, all instances of “sGFP” were incorrectly given as “EGFP”. This error has now been corrected in the PDF and HTML versions of the Article.

