# Supplementary material for: Dynamics of plant DNA replication based on PCNA visualization
Source: Sci Rep. 2016 Jul 15;6:29657. doi: 10.1038/srep29657 (PMC4945867; doi:10.1038/srep29657)
Supplement: Supplementary Information [file srep29657-s1.pdf]

Supplementary information

Dynamics of plant DNA replication based on PCNA visualization

Ryohei Yokoyama[1], Takeshi Hirakawa[1], Seri Hayashi[1], Takuya Sakamoto[1] and Sachihiro Matsunaga[1*]

[1]*Department of Applied Biological Science, Faculty of Science and Technology, Tokyo University of Science, 2641 Yamazaki, Noda, Chiba 278-8510, Japan.*

*For correspondence.

Sachihiro Matsunaga

Department of Applied Biological Science, Faculty of Science and Technology, Tokyo University of Science, 2641 Yamazaki, Noda, Chiba 278-8510, Japan.

Phone: +81-4-7124-1501 Fax: +81-4-7122-9104 E-mail:sachi@rs.tus.ac.jp

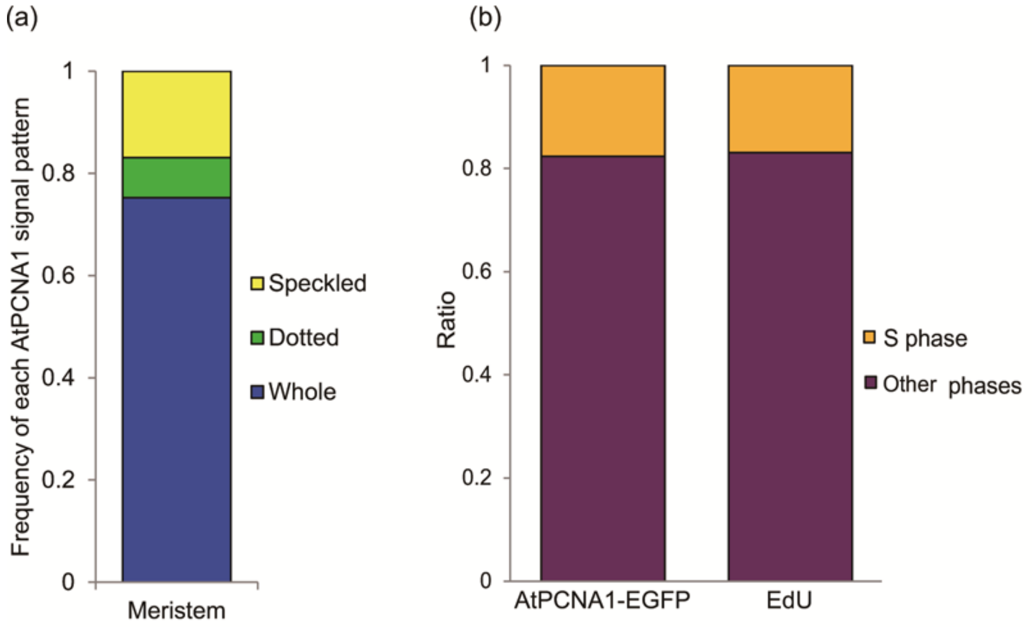

Supplementary Figure S1.

(a) Meristematic epidermal cells were used in AtPCNA1-EdU co-localization analyses (n = 307 nuclei from three plants).

(b) Confirmation of the AtPCNA1-EGFP line as a useful marker line for visualization of S-phase progression. The ratio of the number of nuclei showing AtPCNA1 dotted and speckled patterns to total nuclei was calculated and compared with a previous report using EdU incorporation[6].

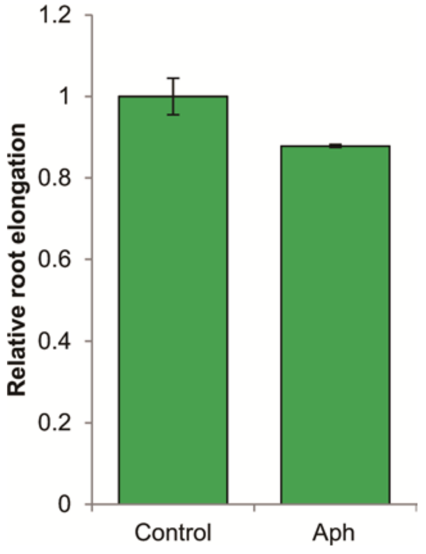

Supplementary Figure S2.

Relative root elongation of pAtPCNA1::AtPCNA1-EGFP on the control medium and medium supplemented with 12 μg/mL aphidicolin. Root length was measured 24 h after transfer to each medium.

Supplementary Table S1. Classification of the pattern of AtPCNA1 and EdU signals in Fig 4.

Type	AtPCNA1 signals	EdU signals
I	whole	whole
II	whole	speckled
III	whole	negative
IV	dotted	whole
V	speckled	whole
VI	speckled	mixed
VII	speckled	speckled

Supplementary Table S2. PCR primers used in this study.

No.	Name	Purpose	Sequence	Tm(℃)
1	AtPCNA1_forward_pENTR_PCR	Cloning	CACCGGGCAAAGTCGGTTTTGGA	64
2	AtPCNA1_reverse_pENTR_PCR	Cloning	GGGATTAGTGTCTTCTTCTTCTTCA	56
3	AtPCNA1 seqence1	Sequencing	GGGCTCGTTGTTGAAGAAGGTTCTA	61
4	AtPCNA1 seqence2	Sequencing	GTAAAACGACGGCCAG	51
5	AtPCNA1 seqence3	Sequencing	GGAAACAGCTATGACCATG	51
